# Process assessment of the attitude, ethics, and communication (AETCOM) sessions: student engagement and satisfaction among medical students in central India

**DOI:** 10.1186/s12909-026-09691-w

**Published:** 2026-06-13

**Authors:** Mamta Dhaneria, Shreya Shrivastava, Reema Shaiva, Ayushi Golecha, Ashish Pathak

**Affiliations:** 1 Department of Pediatrics, Ruxmaniben Deepchand Gardi Medical College, Ujjain, Madhya Pradesh 456006 India; 2 Health Systems and Policy (HSP): Medicines, focusing antibiotics, Department of Global Public Health, Karolinska Institutet, Stockholm, Sweden

**Keywords:** Attitude, Ethics, and Communication (AETCOM), Medical Education, Process Assessment, Competency, System for Evaluation of Teaching Qualities (SETQ)

## Abstract

**Background:**

The Attitude, Ethics, and Communication (AETCOM) module—integral to India’s competency-based medical curriculum—has been designed to foster professionalism, empathy, and ethical decision-making among students graduating from medical colleges in India. However, the process of delivering AETCOM sessions remains insufficiently assessed. Focusing on Phase-III, Part-II MBBS students at R.D. Gardi Medical College, Ujjain, the present study examines how process assessment, based on student feedback and structured observer-based assessment, can provide insights into AETCOM session delivery and student engagement.

**Methods:**

This observational study was conducted on 126 Phase-III, Part-II MBBS students (Batch 2021) over 3.5 months. Four AETCOM competencies—patient autonomy, medical error disclosure, confidentiality, and fiduciary duty—were taught in small groups by using interactive methods, such as role-play and discussions. Data were collected using the modified System for Evaluation of Teaching Qualities (SETQ) survey and an observer-based engagement checklist. The SETQ score ranges from 19 to 95. The actual scores were converted to percentages. Statistical analysis included Cronbach’s alpha for reliability, one-way analysis of variance (ANOVA) for session comparison, and Pearson’s correlation to link the learning environment with student engagement.

**Results:**

The participants’ attendance rate ranged from 92.0% to 99.2%. The modified SETQ demonstrated excellent internal consistency (α = 0.92). Satisfaction scores across the four sessions ranged between 87.97% and 89.86%, categorized as “very good.” A one-way ANOVA revealed significant differences between sessions (*p* = 0.038), with the confidentiality module rated significantly higher than the patient autonomy module. Engagement metrics showed high levels of eye contact (91%–96%) and on-task behavior (84%–94.4%). Pearson correlation showed a strong positive relationship between the facilitator’s professional attitude and student engagement (*r* = 0.70, *p* < 0.01). Note-taking was notably lower (30%–54%), which may reflect greater emphasis on active participation than traditional note-taking.

**Conclusion:**

Process assessment using validated tools like the SETQ provides useful insights into AETCOM session delivery. The findings suggest that interactive methods and positive facilitator–student rapport are associated with higher student engagement. These observations suggest that AETCOM sessions may support student engagement and reflective discussion in ethics-related learning.

**Supplementary Information:**

The online version contains supplementary material available at 10.1186/s12909-026-09691-w.

## Background

The National Medical Commission of India emphasizes the development of essential competencies among medical students to ensure holistic professional growth [1]. To support educators in cultivating these competencies, the Attitude, Ethics, and Communication (AETCOM) module was introduced as a structured, longitudinal program within the competency-based undergraduate medical curriculum [1]. Implemented nationwide from the first professional year through the fourth, the module focuses on core domains such as professionalism, empathy, ethical decision-making, patient autonomy, confidentiality, appropriate disclosure of medical errors, fiduciary responsibility, and effective communication. The AETCOM curriculum comprises 54 competencies, including 39 core competencies and 15 optional conative domain competencies, aimed at shaping ethically grounded and socially responsive Indian medical graduates [1–3].

The AETCOM module has been incorporated into the undergraduate curriculum at R.D. Gardi Medical College, Ujjain. While several studies have evaluated outcomes of AETCOM sessions—such as knowledge gain and skill acquisition—there is limited evidence assessing the delivery process itself [2]. Specifically, aspects such as teaching and learning methods, student engagement, and structured feedback mechanisms have received comparatively little attention, despite their critical role in shaping the learning experience. This lack of process-oriented evaluation represents an important gap, particularly in the context of increasing emphasis on professional conduct and effective patient–doctor communication [3].

To address this gap, R.D. Gardi Medical College introduced a systematic process assessment of AETCOM sessions. This initiative focuses on evaluating teaching methodologies and learning environments through structured student feedback, with the aim of exploring alignment between instructional practices, curricular objectives, and learner perceptions. The present study examines whether such a process-based assessment approach can provide insights into AETCOM session delivery and student engagement thereby contributing to continuous improvement in medical education. The main objectives were to assess student engagement and satisfaction during AETCOM sessions and to evaluate the process of their implementation.

## Methods

### Study design

The present study employed an observational design and was conducted among Phase-III, Part-II MBBS students studying at the Department of Pediatrics, R.D. Gardi Medical College, Ujjain, Madhya Pradesh, from 1st February 2025 to 31st May 2025.

### Sample size

All MBBS students from Batch 2021 (*n* = 126) were included in the study. Further sample size calculation was not required because all students were deemed eligible.

### Data collection method

All Phase-III, Part-II MBBS students from Batch 2021 were considered eligible for the study; however, students who were absent during an AETCOM session were excluded. The principal investigator conducted a session with all eligible participants to explain the study’s purpose. Following this, written informed consent was obtained from the participants.

The following four core AETCOM competencies for students were selected: (a) patient autonomy, patients’ rights, and shared responsibility in healthcare; (b) disclosure of medical errors; (c) confidentiality in patient care; and (d) a doctor’s fiduciary duty. The departmental faculty selected these topics because they are core AETCOM competencies prescribed by the National Medical Commission for Phase III–Part II MBBS students and are highly relevant to day-to-day clinical practice. These competencies—patient autonomy, disclosure of medical errors, confidentiality, and fiduciary duty—commonly arise in real clinical encounters and are well suited for interactive teaching methods such as role-plays and group discussions. The topics were finalized through consensus among departmental faculty to ensure curricular alignment, feasibility of delivery, and meaningful student engagement.

During each session, the attending students (ranging from 113 to 125 per session) were simultaneously divided into three parallel small groups, with 38–42 students in each group, to facilitate interactive learning. Each small group was led by one designated faculty facilitator. The same three facilitators remained consistently assigned to their respective groups across all four AETCOM sessions, and all facilitators followed a standardized teaching plan to ensure uniformity in content delivery and teaching approach.

The four selected AETCOM competencies were delivered through four teaching sessions, with one 60 min session dedicated to each competency. During each session, the attending students (ranging from 113 to 125 per session) were simultaneously divided into three parallel small groups, with 38–42 students in each group, to facilitate interactive learning.

Each small group was conducted in parallel and led by the same designated facilitator for that competency to ensure uniformity in content delivery and teaching approach. Thus, although students participated in small-group discussions, each competency was completed within a single 60 min session, resulting in a total of four AETCOM sessions, and not multiple sequential sessions.

Each session lasted 60 min, starting with a 3–5-minute introduction to the competency topic. This was followed by a 3–5-minute discussion of specific learning objectives. Afterward, a story related to the competency, as outlined in the AETCOM module, was narrated to each small group for 8–10-minute. Then, postgraduate residents from the Department of Pediatrics engaged in a role-play activity for 8–10-minute. The participants were subsequently asked 3–5 pre-defined questions related to each competency domain. For discussion, the entire batch was divided into small groups, each comprising approximately 10 students. The participants discussed answers to these questions after an 8–10-minute buzz session. Then, 3–4 students from each small group presented their opinions. Finally, the facilitator summarized and concluded the discussion, delivering key take-home messages within 5–8-minute.

Following each session, the participants were required to complete a modified System for Evaluation of Teaching Qualities (SETQ) survey [4, 5]. This survey provided immediate feedback on the teaching method, engagement, and overall session quality. The SETQ was modified to include AETCOM-specific questions by replacing two items originally designed for clinical placements. (Supplementary material 1- modified SETQ used in the study).

### Characteristics of the SETQ

The System for Evaluation of Teaching Qualities (SETQ) is a validated instrument developed by Lombarts, M J et al. to assess the teaching performance of clinical educators from the learners’ perspective [5]. The SETQ evaluates key domains of teaching effectiveness, including the development of a positive learning climate, professional attitude toward learners, clear communication of learning objectives, provision of evaluation and constructive feedback, and encouragement of self-directed learning.

The instrument consists of structured statements addressing different aspects of clinical teaching performance. Respondents indicate their level of agreement with each statement using a 5-point Likert scale ranging from 1 (strongly disagree) to 5 (strongly agree). Higher scores reflect better perceived teaching quality. The SETQ has demonstrated strong validity and reliability in evaluating clinical teaching performance in medical and health professional education settings [4, 5].

### Score calculation for the SETQ

For this study, evaluation data for the AETCOM sessions were collected from student responses across four sessions, each focusing on a specific competency: patient autonomy, patient rights, and shared responsibility in healthcare; disclosure of medical error; confidentiality of patient care; and fiduciary duty of a doctor.

The data were analyzed using descriptive statistics to determine the effectiveness of the teaching and learning process based on student feedback across 25 indicators grouped into five categories: teaching and learning environment, professional attitude towards students, communication of goals, evaluation of students, and promoting self-directed learning. Each indicator was rated on a 5-point Likert scale. The total score for each session was calculated, and the percentage score was derived using the formula:

$$\mathrm{Score}\:{(\%)}=\frac{\mathrm{Total}\:\mathrm{score}\:\mathrm{obtained}}{\mathrm{Maximum}\:\mathrm{possible}\:\mathrm{score}}\times100$$

The maximum possible score for each session was calculated as:

Maximum possible score = Number of questions (25) × Number of respondents (N) × Maximum rating (5).

Valuation categories, adapted from Rubiati, were used to classify the percentage scores for each session [6].

### Validation of the SETQ

To ensure alignment with the AETCOM context, two clinical placement questions in the SETQ, adapted from the original instrument were replaced with two AETCOM-specific items [7]. The reliability and internal consistency of the modified SETQ were evaluated using Cronbach’s alpha coefficient (α). A value of α ≥ 0.70 was considered the minimum threshold for acceptable reliability, while values ≥ 0.90 were interpreted as excellent. To ensure content validity, the modified instrument was reviewed by a panel of three medical education experts for relevance, clarity, and suitability for the AETCOM curriculum. Face validity was further confirmed through a pilot session with ten students (not included in the final analysis) to ensure the terminology was unambiguous and culturally appropriate for Phase-III MBBS students in Central India.

### Student engagement checklist

The AETCOM module, as a pivotal component of medical education, have been designed to instill critical competencies in medical students. To assess student engagement during the four AETCOM sessions, an observer-based checklist was employed, focusing on the following five indicators: eye contact, following instructions, on-task behavior, note-taking, and distraction (Supplementary material 2). Student engagement was assessed by four faculty members and ten trained peer assessors (interns), who independently observed approximately 10 randomly selected students per session during small-group discussions. Peer assessors were included to enhance the objectivity and reliability of engagement assessment. Independent observations by both faculty and peer assessors helped reduce observer bias. The checklist used for quantifying student engagement was based on a binary scoring system. Specifically, for eye contact, following instructions, on-task behavior, and note-taking, a “Yes” response earned + 1 point, indicating active engagement, whereas a “No” response was assigned ˗1 point, reflecting a lack of engagement. Conversely, for distraction, a “No” response earned + 1 point, indicating minimal off-task behavior, whereas a “Yes” response was given ˗1 point, denoting disruptive actions. The percentage score for each indicator was calculated as the proportion of positive responses relative to the total number of respondents, providing a standardized measure of engagement.

### Ethical considerations

The study was approved by the Institutional Ethics Committee (reference number IEC-07/2025). Since MBBS students are considered a vulnerable population, all students were assured that participation in the study would not affect their assessment in the subject of Pediatrics. Furthermore, written informed consent was obtained before data collection, and confidentiality and anonymity of the students were maintained. Participation was voluntary, and students could withdraw from the study at any time without consequences. The procedures followed were in accordance with the ethical standards set by the Institutional Ethics Committee and the Declaration of Helsinki.

### Statistical analysis

Data from students were recorded on paper forms and digitized using Microsoft Excel for statistical processing. Continuous data were expressed as mean ± standard deviation and range. Categorical variables were presented as percentages. Comparisons between two groups were performed using the independent t-test for continuous variables and the Chi-square test or Fisher’s exact test for categorical variables, as appropriate.

To explore the relationship between the learning environment and student participation, Pearson’s correlation coefficients (r) were calculated between the five domains of the modified SETQ and the indicators of the Student Engagement Checklist. The strength of the correlation was interpreted as weak (*r* < 0.3), moderate (0.3–0.7), or strong (*r* > 0.7).

To compare the effectiveness of the four AETCOM competency sessions, a one-way analysis of variance (ANOVA) was performed on the SETQ percentage scores. Before the analysis, the assumption of normality was verified using the Shapiro-Wilk test, and homogeneity of variances was confirmed using Levene’s test. In cases where the ANOVA indicated significant differences (*p* < 0.05), Tukey’s Honestly Significant Difference (HSD) post-hoc test was employed to identify specific pairs of sessions that differed. All psychometric and correlational analyses were performed using Stata 16.0 (StataCorp LLC, College Station, TX, USA), with a p-value of < 0.05 considered statistically significant. Data visualization was performed using GraphPad Prism 7 (GraphPad Software, San Diego, CA, USA).

### Handling of the Hawthorne effect

As this study involved observation of student engagement and immediate post-session feedback, the potential influence of the Hawthorne effect—where participants alter behavior due to awareness of being observed—was considered during study design.

To minimize this effect, the AETCOM sessions were conducted as part of the routine academic schedule rather than as special research activities, thereby reducing novelty-related behavioral changes. Observations during small-group discussions were non-intrusive, with observers maintaining a passive role and without disclosing specific behavioral indicators being assessed. Engagement was evaluated across four sessions over 3.5 months, allowing any initial reactivity to diminish over time.

Student feedback through the modified SETQ was collected anonymously, and participants were assured that their academic assessment would not be affected, reducing socially desirable responses. Engagement data were analyzed at the aggregate level rather than individually to minimize performance pressure.

## Results

**Fig. 1:**
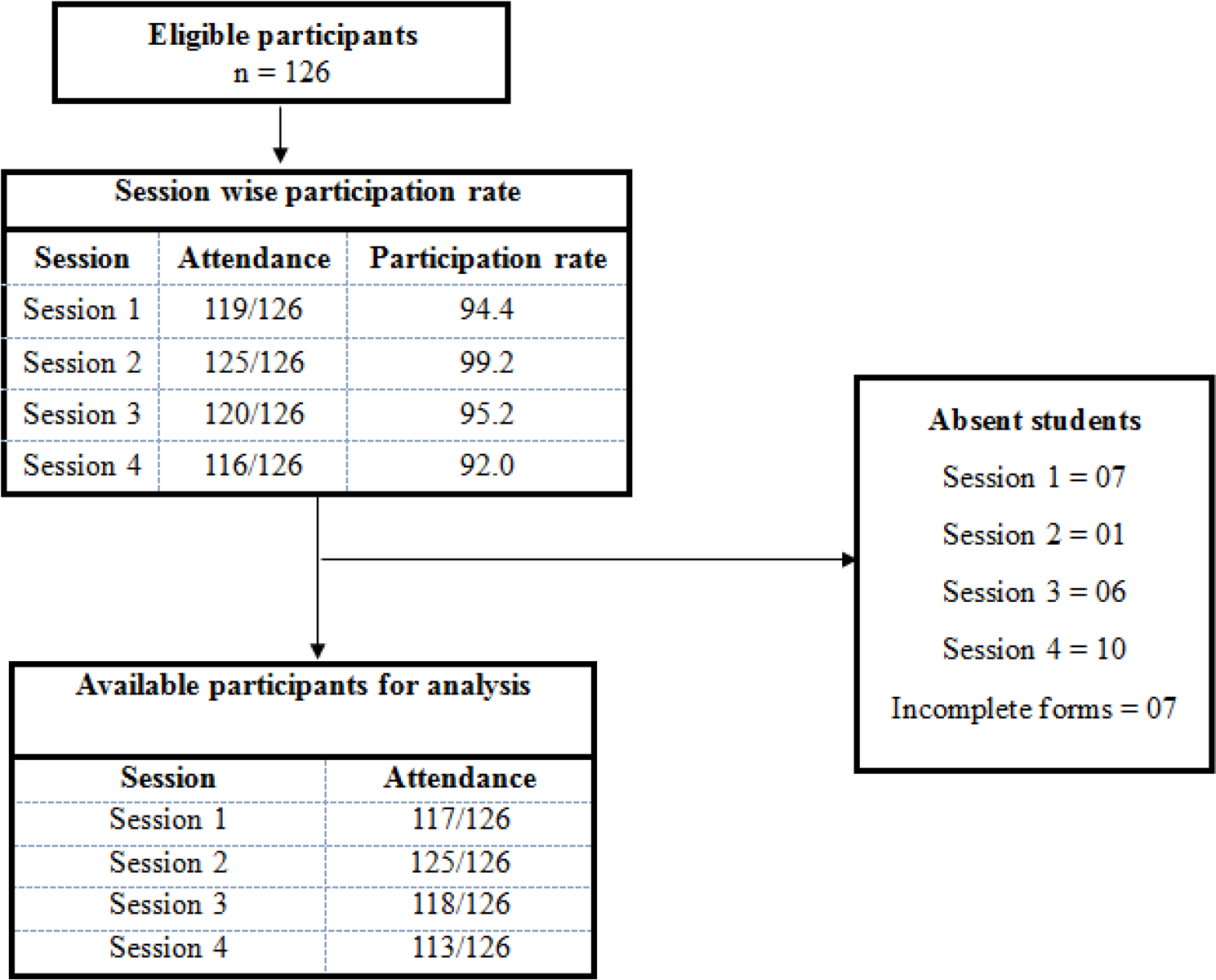
Flow chart of recruitment of participants

A total of 126 participants were enrolled for the study. Session-wise attendance of students was 117 (94.4%), 125 (99.2%), 118 (95.2%) and 113 (92%) respectively. The maximum attendance was in session 2. Figure 1 presents the flow chart of participant recruitment.

The reliability of the modified SETQ was assessed using Cronbach’s alpha coefficient (α) to ensure internal consistency within the Indian medical education context. The overall scale demonstrated excellent reliability (α = 0.92). Sub-scale reliability ranged from 0.79 to 0.89 (Table 1), indicating that the instrument consistently measured the intended dimensions of the affective domain learning environment across all four AETCOM modules.

**Table 1:** The reliability of the modified SETQ

Scale / Sub-scale	No. of Items	Cronbach’s Alpha (α)	Internal Consistency
Total SETQ Scale	25	0.92	Excellent
1. Teaching and Learning Environment	6	0.86	Good
2. Professional Attitude	4	0.84	Good
3. Communication of Goals	3	0.79	Acceptable
4. Evaluation and Feedback	9	0.89	Good
5. Self-Directed Learning	3	0.81	Good

Table 2 presents student feedback on facilitator activities across 6 key domains, comprising 25 questions, pertaining to the competency of patient autonomy, rights, and shared healthcare responsibility.

**Table 2:** Students’ evaluation of the AETCOM session-1 using SETQ scale for the competency-patient’s autonomy, patient’s rights, and shared responsibility in healthcare

S.no	Activities	*n*	Valuation					Total score obtained	Possible maximum score
			1	2	3	4	5		
Teaching and Learning environment									
Q1.	Encourages students to participate actively in discussions	117	0	0	7	49	61	522	585
Q2.	Stimulates students to bring up problems	117	0	2	6	59	50	508	585
Q3.	Keeps to teaching goals; avoids digressions	117	0	2	12	61	42	494	585
Q4.	Prepares well for teaching presentations and talks	117	0	2	3	41	71	532	585
Q5.	Teaches the topic in theory and practical	117	0	2	12	40	63	515	585
Q6.	Covering all the points in the topic	117	0	0	4	51	62	526	585
Professional attitude towards students									
Q7.	Listens attentively to students	117	0	0	5	46	66	529	585
Q8.	Is respectful towards students	117	0	0	4	45	68	532	585
Q9.	Is available regularly for the students	117	0	3	9	48	57	510	585
Q10.	Is easily approachable for discussions	117	0	0	6	52	59	521	585
Communication of Goals									
Q11.	States learning goals clearly	117	0	1	8	58	50	508	585
Q12.	Prioritizes learning goals and topics	117	0	1	6	54	56	516	585
Q13.	Debriefing the learning goals periodically	117	0	2	12	55	48	500	585
Evaluation of Students									
Q14.	Evaluates student’s specialty knowledge regularly	117	0	1	15	56	45	496	585
Q15.	Evaluates student’s analytical abilities regularly	117	0	0	13	58	46	501	585
Q16.	Evaluates student’s application of knowledge to specific patients	117	1	0	12	50	54	507	585
Q17.	Evaluates student’s medical skills regularly	117	0	1	10	56	50	506	585
Q18.	Evaluates student’s, communication and professionalism during patient encounter	117	0	1	8	52	56	514	585
Feedback									
Q19.	Regularly gives constructive feedback to students	117	0	3	9	56	49	502	585
Q20.	Explains why students are incorrect	117	0	1	6	57	53	513	585
Q21.	Offers suggestions for improvement	117	0	0	3	52	62	527	585
Q22.	Gives students chance to reflect on the feedback	117	0	0	3	53	61	526	585
Promoting self-directed learning									
Q23.	Motivates students to study further and deeper in the topic	117	0	1	5	53	58	519	585
Q24.	Stimulates students to keep up with the literature	117	0	1	5	51	60	521	585
Q25.	Motivates students to learn independently	117	0	2	8	40	67	523	585
			1	26	191	1293	1414	12,868	14,625

Score Calculation:

$$\mathrm{Score}\:(\%)=\frac{\mathrm{Total}\:\mathrm{Score}\:\mathrm{obtained}}{\mathrm{Maximum}\:\mathrm{Possible}\:\mathrm{score}}\times100$$

- Total score obtained = 12,868.
- Maximum possible score = 25 questions × 117 respondents × 5 = 14,625.

$$\mathrm{Score}\:(\%)=\frac{12,868}{\mathrm{14,625}}\times100\:=\:87.97\:{\%}\:(\mathrm{very}\:\mathrm{good})$$

Overall, students reported positive perceptions across all SETQ domains, including teaching and learning environment, professional attitude, communication of goals, evaluation and feedback, and promotion of self-directed learning (Table 2). Across the four AETCOM sessions, facilitators were consistently rated highly for respectful behavior, attentive listening, encouragement of active participation, clear communication of learning objectives, constructive feedback, and promotion of independent learning. Students particularly appreciated opportunities for reflective feedback and communication during patient encounters. Minor variations were observed between sessions, with the confidentiality module receiving comparatively higher ratings for facilitator preparedness and communication of goals (Supplementary Tables 1–3). These findings indicate a consistently favorable learning environment with high levels of student engagement and satisfaction across all sessions.

Table 3 summarizes the total SETQI scores for the four AETCOM sessions, demonstrating consistently high evaluation scores, all rated as “Very good.”

**Table 3:** Summary of evaluation of the four AETCOM sessions using SETQ by the students

S.no	Competency	Respondents (*n*)	Total Score	Maximum Score	Score (%)	Valuation
1.	Patient autonomy	117	12,868	14,625	87.97	Very good
2.	Disclosure of medical error	125	13,904	15,625	88.99	Very good
3.	Confidentiality in patient care	118	13,254	14,750	89.86	Very good
4.	Fiduciary duty of a doctor	113	12,677	14,125	89.73	Very good

**Fig. 2:**
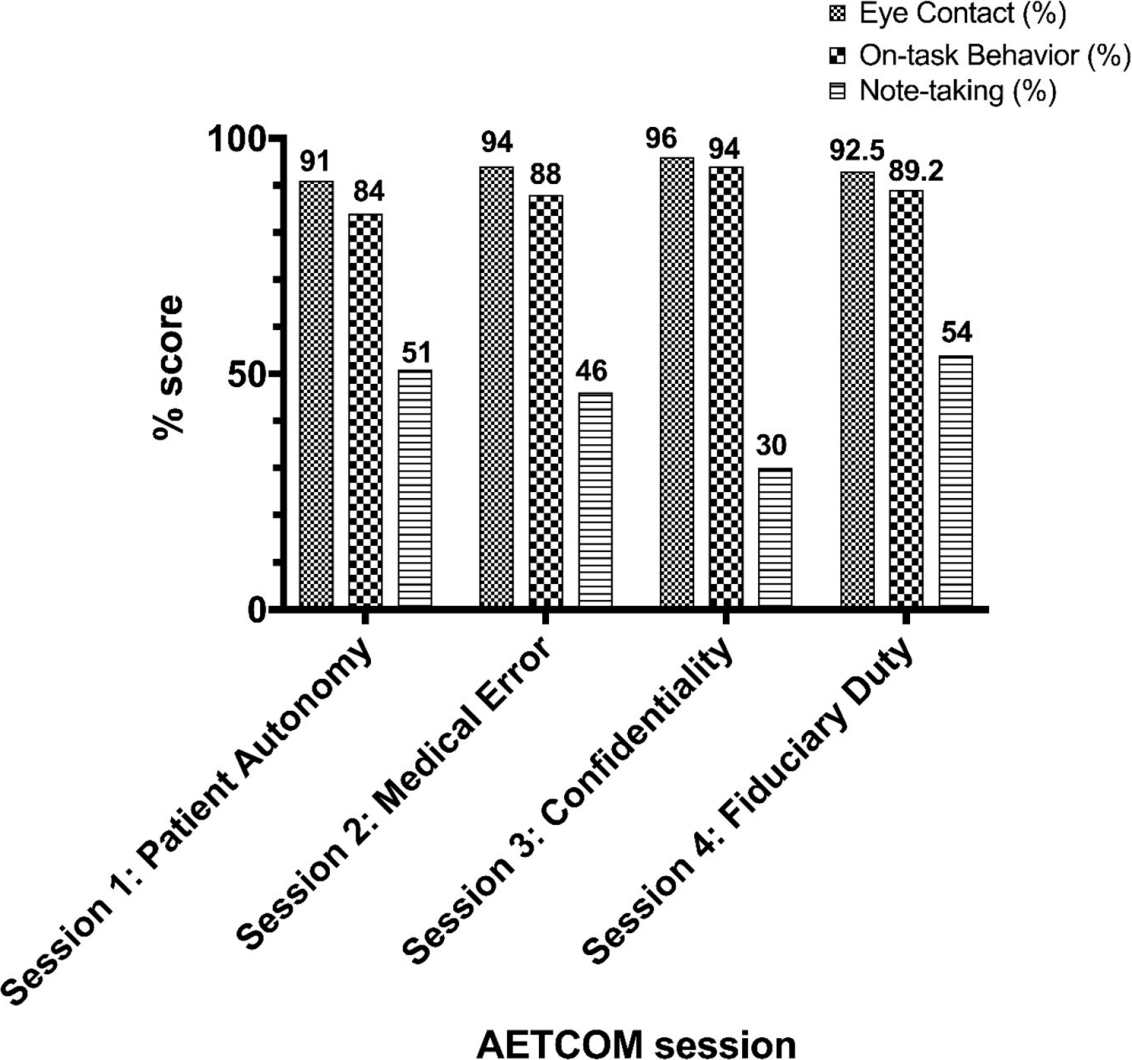
Comparison of active and passive engagement metrics across AETCOM sessions

The bar chart illustrates the percentage of students demonstrating specific engagement behaviors during four AETCOM sessions (Fig. 2). A significant behavioral pattern was observed in student engagement across all competencies (Fig. 2). While students maintained exceptionally high levels of eye contact (reaching 96.0% in the confidentiality session) and on-task behavior, there was a marked decline in note-taking. This was most prominent in Session 3 (confidentiality), where only 30% of students engaged in writing. This may indicate that the interactive nature of the AETCOM sessions was associated with greater participation in dialogue and reflection, reducing the perceived need for traditional passive documentation.

A Pearson correlation analysis was conducted to examine the relationship between the SETQ and observed student engagement (Table 4).

**Table 4:** Correlation between SETQ domains and student engagement metrics (Pearson’s r)

SETQ Domain	Eye Contact	Following Instructions	On-Task Behavior
Teaching and Learning Environment	0.68*	0.59*	0.62*
Professional Attitude	0.74**	0.65*	0.70**
Communication of Goals	0.55*	0.72**	0.58*
Evaluation and Feedback	0.61*	0.60*	0.66*
Self-Directed Learning	0.52*	0.48	0.54*

*p < 0.05; **p < 0.01

There was a significant positive correlation between the facilitator’s professional attitude and students’ on-task behavior (*r* = 0.70, *p* < 0.01). Furthermore, communication of goals was strongly correlated with students following instructions (*r* = 0.72, *p* < 0.01). These findings indicate positive correlations between student engagement metrics and perceived quality of the instructional environment and facilitator conduct.

The one-way ANOVA revealed a statistically significant difference in the SETQ satisfaction scores across the four sessions, *F* (3, 469) = 2.84, *p* = 0.038 (Table 5).

**Table 5:** Comparison of SETQ mean percentage scores across four AETCOM sessions

AETCOM Session Topic	*N*	Mean Score (%)	Std. Deviation	F-statistic	*p*-value
1. Patient Autonomy	117	87.97	4.21	2.84	0.038*
2. Medical Error Disclosure	125	88.99	3.85		
3. Confidentiality	118	89.86	3.42		
4. Fiduciary Duty	113	89.73	3.98		

*Significant at *p* < 0.05 level

Post-hoc analysis (Tukey HSD) indicated that the session on confidentiality in patient care was rated significantly higher by students than the session on patient autonomy (*p* < 0.05). No significant differences were found between sessions 2, 3, and 4. This suggests that while all sessions were “very good,” the structured delivery of confidentiality session appeared to be particularly well received by this cohort.

**Fig. 3:**
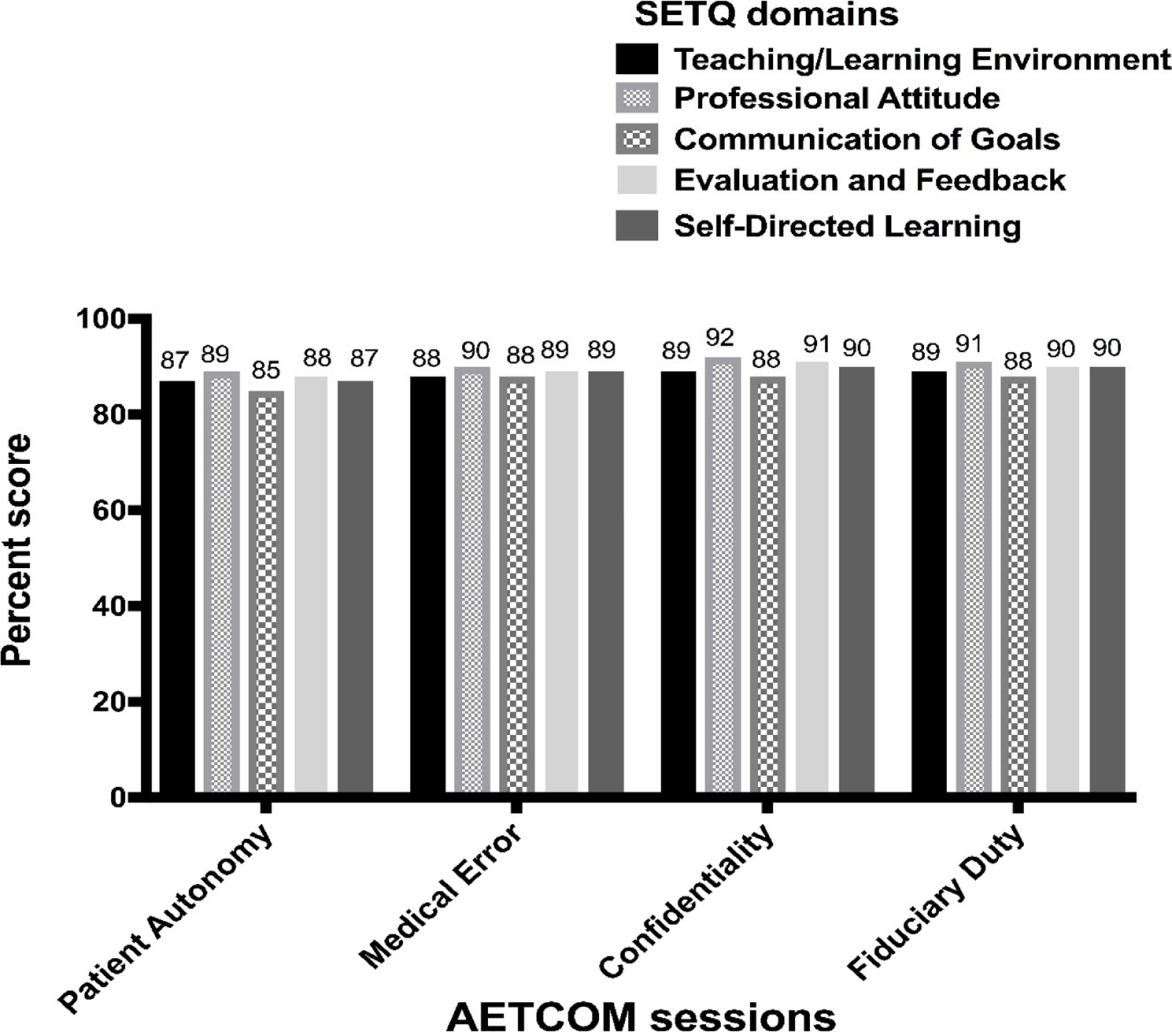
Bar chart showing mean SETQ domain scores across four teaching and learning sessions

The comparative performance of the four AETCOM modules across the five SETQ domains is visualized in Fig. 3. The bar chart shows that the delivery of the AETCOM curriculum was consistent regardless of the specific competency being taught. This visualization suggests relative consistency of SETQ domain scores across sessions.

## Discussion

India supplies a substantial proportion of health professionals worldwide, contributing immensely to global healthcare [8]. This global contribution has necessitated a curriculum meticulously aligned with professional attributes that are locally pertinent as well as globally adaptable [9]. While this critical realization has profoundly resonated with health professionals nationwide, comprehensive efforts to address this issue have not been made and remain insufficiently articulated. Historically, in the medical curriculum, rigorous teaching and the integration of vital domains, such as medical ethics, behavioral science, communication skills, and managerial competencies, have been overlooked [10]. Embodying the previously overlooked aspects, the proposed AETCOM module comprehensively integrates and balances the following five crucial roles required to be played by an “Indian Medical Graduate”: clinician; leader and member of the healthcare team; communicator; lifelong learner; and professional [11]. Learning in the affective domain was hitherto relegated to the hidden curriculum, which poses unique challenges, and requires the use of additional teaching and learning methods [12]. Attitude, communication, and ethics are the key domains that need to be taught directly and explicitly to undergraduates in their curriculum [12].

Overall, all sessions were highly appreciated, with scores clustered between 87.97% and 89.86%. Statistical comparison using one-way ANOVA revealed a significant difference between sessions (*p* = 0.038). Confidentiality in patient care (AETCOM session-3) received the highest mean SETQ scores care (AETCOM session-3), significantly outperforming patient autonomy (AETCOM session-1) [11–13]. This suggests that while the overall delivery was well received by students, the immersive nature of confidentiality-based role-plays may have resonated more deeply with students or created a perceived gap in their prior ethical training. The high attendance rate and consistently positive feedback across all sessions underscore the high level of student engagement and satisfaction associated with the chosen teaching methodologies [13]. The significance of these modules is further substantiated by students’ positive responses concerning empathy and communication skills consistently [13]. Studies examining ethics and communication modules have similarly reported high student satisfaction and engagement in areas such as knowledge of patient rights, communication abilities, and development of professional responsibilities [13]. For instance, dedicated ethics and communication courses were reported to be associated with improved student understanding of medical law and reduce anxiety surrounding potential medical disputes [14]. Students have demonstrated interest in formal training in ethics and communication, and such sessions were perceived as beneficial for cultivating empathy and supporting better patient interactions by over 85% of participants in a study [15].

Our findings on student engagement suggested that participants prioritized active discussion and participation over traditional note-taking [16–18]. While traditionally, note-taking is linked to memorization and retention of factual knowledge, AETCOM sessions are primarily designed for internalizing values, developing empathy, and fostering ethical decision-making, which are cultivated through active participation and reflection rather than extensive rote learning. Therefore, the observed lower rates of note-taking are consistent with the module’s objective of promoting active participation and reflective learning rather than the mere recall of information. Critically, Pearson correlation analysis demonstrated that this engagement was not random; a strong positive correlation (r = 0.70, p < 0.01) was found between the facilitator’s ‘Professional Attitude’ and students’ ‘On-task Behavior.’ This suggests that higher professional attitude scores were positively correlated with eye contact (91%–96%) and on task behavior.

In the present study, the SETQ was used to evaluate teaching quality. While the SETQ has been validated and utilized globally for evaluating teaching quality [4], our analysis supports its applicability in this setting with a Cronbach’s alpha of 0.92, indicating excellent internal consistency. This finding aligns with other validation studies that demonstrate strong reliability and a clear multi-dimensional structure for SETQ. SETQ has been used to evaluate surgical training by surgery and gynecology residents [16], pediatric urology residents [17], neurosurgery residents [18], and and among anesthesiology residents in a multicenter study [19]. It has also been used for evaluation of teaching quality in undergraduate education with high internal consistency of five subscales.

The psychometric properties of the Persian version of SETQ has been evaluated by post-graduate [20] and graduate medical students [4]. Furthermore, the Exploratory Factor Analysis performed in this study validated the structural integrity of the five domains, confirming that the tool accurately captures the multifaceted nature of the AETCOM learning environment [11, 21]. This approach is vital given the shift to competency-based medical education, which prioritizes professionalism and core competencies, in India [22].

The findings of the present study indicate that the SETQ is a sensitive tool for capturing variations in students’ perceptions of the learning environment. In the context of competency-based medical education in India, which emphasizes learner-centered and outcome-based approaches, tools like SETQ can offer valuable insights into domains such as professionalism, communication, and ethical competencies addressed through the AETCOM module [2, 10, 22, 23].

Differences in SETQ scores between AETCOM sessions (Sessions 1–4) were observed (Table 3), warranting consideration of facilitator influence. However, instructor-related variability was minimized in the present study because the same three facilitators consistently conducted their respective small groups across all four competency sessions using standardized teaching materials, learning objectives, role-play structure, discussion prompts, and take-home messages. Therefore, the observed statistically significant differences are more plausibly attributable to intrinsic topic-related factors such as perceived clinical relevance, ethical complexity, or the immersive nature of specific case scenarios—particularly in the confidentiality module. These findings suggest that thematic engagement and contextual relatability may have contributed to differences in student perceptions and participation during affective-domain teaching within competency-based medical education.

Studies have reported that the faculty across medical colleges in India face challenges in teaching and assessing the affective domain [15, 24, 25]. By demonstrating high sensitivity through the SETQ and correlating it with objective engagement checklists, our study suggests a dual-assessment framework that highlights the inherent subjectivity of Miller’s pyramid in ethics education. Owing to limited training in applying the methods for teaching and learning and assessment of the affective domain. Learning objectives in the affective domain, when assessed, are taken more seriously by both teachers and students. In the absence of such assessments, students may graduate without being trained in the affective domain, leading to suboptimal patient care [26, 27]. In the present study, specified behaviors were carefully observed by multiple observers—a prerequisite for assessment of the affective domain [28]. Miller’s pyramid is a widely used 4-level framework for such assessment [29]. However, adapting this framework for the nuanced evaluation of attitudes, ethics, and communication within the AETCOM module presents unique challenges due to the subjective nature of these competencies and the limitations of traditional assessment tools.

The implementation of the AETCOM module in the undergraduate medical curriculum in India highlights the critical need to move beyond traditional knowledge-centric education and ensure explicit teaching of essential competencies that constitute the affective domain [15, 30]. This shift mandates a paradigm change in pedagogical strategies, emphasizing experiential learning and reflective practices to cultivate professionalism and empathy among future medical practitioners [10, 31]. The evolving curriculum design directly accounts for the recognized deficiencies in past medical education, which prioritized cognitive and psychomotor domains, while neglecting the development of empathy, communication, and ethical reasoning among medical practitioners [13].

Comprehensive understanding of the perceptions of students and faculty members remains crucial for understanding and informing the implementation of the AETCOM module within the evolving medical education landscape [32]. To address these challenges and ensure uniform implementation, institutions must conduct faculty training programs, particularly those on competency-based medical education and diverse teaching and learning methods for the affective domain [32].

### Strengths and limitations

Our study has several key strengths. It effectively addresses a notable gap by focusing on the process assessment of AETCOM modules, rather than just their outcomes. The research employs a robust methodology, utilizing a modified SETQ scale which demonstrated excellent internal consistency (α = 0.92). High student satisfaction (ranging from 87.97% to 89.86%) and engagement were consistently observed, driven by interactive pedagogical methods like role-plays and discussions. Furthermore, the study identified a significant positive correlation between the facilitator’s professional attitude and student engagement, providing preliminary insights into factors associated with student engagement during AETCOM sessions.

The present study has several limitations, including its single-center design, which restricts generalizability. In addition, the study primarily assessed the delivery process, rather than the long-term impact of AETCOM modules on clinical practice, thus offering limited insight into sustained professional conduct. Furthermore, the inherent subjectivity of affective domain competencies (attitudes, ethics, and communication) posed challenges for objective measurement, despite efforts to adapt the assessment tools. The ‘Student Engagement’ checklist was not adopted from any source. It was made by the faculty members of the department and is not validated. Although the Hawthorne effect cannot be eliminated in educational research, measures mentioned in the methods section were implemented to enhance the validity of the findings. Finally, variability in faculty preparedness for teaching and evaluating this domain could have influenced session fidelity and outcomes.

## Conclusions

The integration of the AETCOM module into the undergraduate medical curriculum represents an important curricular initiative toward producing ethically sound and empathetic Indian Medical Graduates. This study indicates that the quality of the clinical learning environment, as measured by the validated SETQ instrument, is consistently high and is positively associated with student engagement levels. Key findings indicate that interactive pedagogical methods—such as role-plays and buzz sessions—were associated with high student satisfaction (87.97%–89.86%) and active participation during sessions. The significant positive correlation (*r* = 0.70) between facilitator professional attitude and student engagement highlights the potential importance of facilitator rapport and preparedness in supporting student engagement. Furthermore, the high internal consistency (α = 0.92) of the SETQ may provide a useful framefork for assessing student perceptions of the learning environment. While all ethical competencies were rated as “very good,” the higher student ratings for the confidentiality session may reflect greater student engagement with immersive scenarios in this setting. Future efforts should focus on multi-center validation and longitudinal studies to evaluate how whether these perceptions and engagement patterns translate into sustained professional conduct in clinical practice. Ultimately, prioritizing the systematic assessment of the teaching and learning process can provide insights into the implementation of the AETCOM module in medical education.

## Supplementary Information

Supplementary Material 1.

Supplementary Material 2.

Supplementary Material 3.

Supplementary Material 4.

Supplementary Material 5.

## Data Availability

The dataset used and/or analysed during the current study is available from the corresponding author on reasonable request. Individual data can due to confidentiality reasons not be made public. All enquiries regarding data sharing should be made to- The Chairman, Institutional Ethics Committee, R D Gardi Medical College, Agar Road, Ujjain, India 456006. The name of data set corresponding to the study is AETCOM data.

## Abbreviations

AETCOM: Attitude, Ethics, and Communication
CBME: Competency-Based Medical Education
IEC: Institutional Ethics Committee
MBBS: Bachelor of Medicine and Bachelor of Surgery
NMC: National Medical Commission
SETQ: System for Evaluation of Teaching Qualities
SD: Standard Deviation
TL: Teaching and Learning

## References

[1] National Medical Commission. Attitude, Ethics and Communication (AETCOM) module. New Delhi: National Medical Commission. 2020. Available from: https://www.nmc.org.in/wp-content/uploads/2020/01/AETCOM_book.pdf. Accessed 28 Jun 2025.

[2] Abeetha SSA, Suma S, Sebastian S, Chitra M, Shruthy KM. Assessment of Knowledge, Attitude and Perception on AETCOM module- Empathy and Communication Skills among Phase I medical students. Afr J Biomed Res. 2024;27(4s):3717–22.

[3] Stephenson CR, Bonnes SL, Sawatsky AP, Richards LW, Schleck CD, Mandrekar JN, Beckman TJ, Wittich CM. The relationship between learner engagement and teaching effectiveness: a novel assessment of student engagement in continuing medical education. BMC Med Educ. 2020;20(1):403. 33148231 10.1186/s12909-020-02331-x PMC7640708

[4] Al Ansari A, Strachan K, Hashim S, Otoom S. Analysis of psychometric properties of the modified SETQ tool in undergraduate medical education. BMC Med Educ. 2017;17(1):56. 28302151 10.1186/s12909-017-0893-4 PMC5356325

[5] Lombarts MJ, Arah OA, Busch OR, Heineman MJ. [Using the SETQ system to evaluate and improve teaching qualities of clinical teachers]. Ned Tijdschr Geneeskd. 2010;154:A1222. 20170574

[6] Rubiati R. Research of improving students’ speaking skill through debate technique [thesis]. Indonesia: [Semarang: Walisongo State Institute for Islamic Studies]; 2010. Available from https://eprints.walisongo.ac.id/3414.

[7] Salamonson Y, Bourgeois S, Everett B, Weaver R, Peters K, Jackson D. Psychometric testing of the abbreviated Clinical Learning Environment Inventory (CLEI-19). J Adv Nurs. 2011;67(12):2668–76. 21722165 10.1111/j.1365-2648.2011.05704.x

[8] Hawkes M, Kolenko M, Shockness M, Diwaker K. Nursing brain drain from India. Hum Resour Health. 2009;7:5. 19187559 10.1186/1478-4491-7-5 PMC2642753

[9] Zodpey S, Lumbiganon P, Evans T, Yang K, Ha BTT, Negandhi H, Chuenkongkaew W, Al-Kabir A. Assessment of health professional education across five Asian countries-a protocol. Hum Resour Health. 2018;16(1):52. 30285862 10.1186/s12960-018-0316-6 PMC6171128

[10] Chen G, Wang H, Zhou L, Yang J, Xu L, Liang Y. Development and applications of graduate outcome-based curriculum for basic medical education. Front Med (Lausanne). 2024;11:1400811. 39219793 10.3389/fmed.2024.1400811 PMC11362034

[11] Udgiri R, Ganganahalli P. Perceptions of Attitude, Ethics, and Communication (AETCOM) Modules Among Indian Medical Graduates in Their First Professional Year: An Educational Observational Study. Cureus. 2024;16(7):e64611. 39149658 10.7759/cureus.64611 PMC11326756

[12] Singh S, Dhaliwal U, Singh N. Developing Humanistic Competencies Within the Competency-Based Curriculum. Indian Pediatr. 2020;57(11):1060–6. 32893828 10.1007/s13312-020-2036-y PMC7678630

[13] Abeetha SSA, Suma S, Stephy Sebastian M, Chitra KM, Shruthy. Assessment of Knowledge, Attitude and Perception on AETCOM module- Empathy and Communication Skills among Phase I medical students. 3717. 10.53555/ajbr.v27i4s.4171 *Afr J Biomed Res* 2024, 27(4s): 3717–3722.

[14] Chen WT, Fu CP, Chang YD, Shiao YC, Chen PY, Wang CC. Developing an innovative medical ethics and law curriculum-constructing a situation-based, interdisciplinary, court-based learning course: a mixed methods study. BMC Med Educ. 2022;22(1):284. 35428246 10.1186/s12909-022-03349-z PMC9011998

[15] Govindraj L, Santhosh S, Sunish SC, Gopalakrishnan AV, Chandy SJ, Oommen V, Shanthi Fx M. Effectiveness of teaching medical ethics to medical students on an online platform: An analysis of students’ perceptions and feedback. Indian J Med Ethics. 2023;VIII(1):32–8. 35699300 10.20529/IJME.2022.034

[16] Lases SS, Arah OA, Pierik EG, Heineman E, Lombarts MJ. Residents’ engagement and empathy associated with their perception of faculty’s teaching performance. World J Surg. 2014;38(11):2753–60. 25008244 10.1007/s00268-014-2687-8

[17] Silverii H, Ahn J, Gopalan M, Lendvay TS, Gupta A, Kieran K, Shnorhavorian M, Joyner B, Cain MP, Merguerian P, et al. Evaluating the impact of a faculty focused coaching model on surgical training in pediatric urology. J Pediatr Urol. 2026;22(2):105732. 41671639 10.1016/j.jpurol.2026.105732

[18] Yong RL, Cheung W, Shrivastava RK, Bederson JB. Teaching quality in neurosurgery: quantitating outcomes over time. J Neurosurg. 2022;136(4):1147–56. 34479202 10.3171/2021.2.JNS203900

[19] Lombarts KM, Ferguson A, Hollmann MW, Malling B, Collaborators S, Arah OA. Redesign of the System for Evaluation of Teaching Qualities in Anesthesiology Residency Training (SETQ Smart). Anesthesiology. 2016;125(5):1056–65. 27606931 10.1097/ALN.0000000000001341

[20] Boldaji FT, Amini M, Parvizi MM. Psychometric properties of the Persian version of System for Evaluation of Teaching Qualities by students: A tool for assessing clinical tutors from students’ viewpoint. J Educ Health Promot. 2022;11:92. 35573607 10.4103/jehp.jehp_1622_20 PMC9093631

[21] Lal S, Sehgal P. Integration of Attitude, Ethics, and Communication Competencies into Competency-based UG Curriculum. Indian J Community Med. 2022;47(1):4–7. 35368474 10.4103/ijcm.ijcm_1022_21 PMC8971870

[22] Sahanaa C, Niranjan R, Pradeep K, Gopinath S, Dhanasekar E, Vendhan S, Maniradjou V, Konduru RK, Phalsalkar M. Innovative AETCOM session on health care as a right: Experience at the medical college in Puducherry. J Educ Health Promot. 2023;12:386. 38333150 10.4103/jehp.jehp_267_23 PMC10852154

[23] Shanmugam JRR, Kumar M, Gopalakrishna SM, Palanisamy KT, Narayanan S. Perspectives of Teachers at Medical Colleges Across India regarding the Competency based Medical Education Curriculum – A Qualitative, Manual, Theoretical Thematic Content Analysis. Indian J Community Health Indian J Community Health. 2023;35(1):32.

[24] D’Souza C, Jain A, Mandal T, Mohammed CA, Hl KP, Pinto S. An educational approach using interprofessional (IP) role plays and patient narratives to inculcate empathy and communication among undergraduates in breast cancer management. BMC Med Educ. 2024;24(1):1320. 39550590 10.1186/s12909-024-05997-9 PMC11568588

[25] Jain T, Mohan Y, Maiya GR, Nesan G, Boominathan C, Eashwar AVM. Evaluating the effectiveness of ‘AETCOM Module’ on the medical interns posted in peripheral health centres of a tertiary care medical college in Kanchipuram, Tamil Nadu. J Family Med Prim Care. 2022;11(6):2828–33. 36119158 10.4103/jfmpc.jfmpc_1647_21 PMC9480674

[26] Yuen JK, See C, Cheung JTK, Lum CM, Lee JS, Wong WT. Can teaching serious illness communication skills foster multidimensional empathy? A mixed-methods study. BMC Med Educ. 2023;23(1):20. 36631787 10.1186/s12909-023-04010-z PMC9835381

[27] Zhang Z, Hu Q, Xu C, Zhou J, Li J. Medical teachers’ affective domain teaching dilemma and path exploration: a cross-sectional study. BMC Med Educ. 2022;22(1):883. 36539780 10.1186/s12909-022-03870-1 PMC9764310

[28] Zhou YC, Tan SR, Tan CGH, Ng MSP, Lim KH, Tan LHE, Ong YT, Cheong CWS, Chin AMC, Chiam M, et al. A systematic scoping review of approaches to teaching and assessing empathy in medicine. BMC Med Educ. 2021;21(1):292. 34020647 10.1186/s12909-021-02697-6 PMC8140468

[29] Witheridge A, Ferns G, Scott-Smith W. Revisiting Miller’s pyramid in medical education: the gap between traditional assessment and diagnostic reasoning. Int J Med Educ. 2019;10:191–2. 31655795 10.5116/ijme.5d9b.0c37 PMC7246123

[30] Chandra Shaw S, Datta K, Lall M, Jaipurkar R, Shakya AK, Kanitkar M. Development of a hybrid undergraduate portfolio for the AETCOM module. Med J Armed Forces India. 2024;80(2):192–8. 38525458 10.1016/j.mjafi.2022.04.001 PMC10954497

[31] Chai X, Yang J, Liu Y. Influential factors for medical students’ classroom concentration-evaluation with speech recognition and face recognition technology. BMC Med Educ. 2024;24(1):1236. 39478559 10.1186/s12909-024-06204-5 PMC11526676

[32] Ramanathan R, Shanmugam J, Sridhar MG, Palanisamy K, Narayanan S. Exploring faculty perspectives on competency-based medical education: A report from India. J Educ Health Promot. 2021;10:402. 34912938 10.4103/jehp.jehp_1264_20 PMC8641753

