# Supplementary material for: Process assessment of the attitude, ethics, and communication (AETCOM) sessions: student engagement and satisfaction among medical students in central India
Source: BMC Med Educ. 2026 Jun 13;26:963. doi: 10.1186/s12909-026-09691-w (PMC13263919; doi:10.1186/s12909-026-09691-w)
Supplement: Supplementary file 1 — Supplementary Material 1. [file 12909_2026_9691_MOESM1_ESM.docx]

**
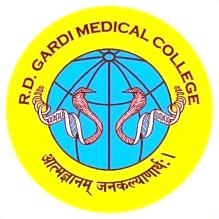
**

**AETCOM SESSIONS**

**Evaluation of Clinical Tutor by Students**

Student Name: _______________________ Date of Class: ______________

Student ID: ____________

Teacher Name: _______________________

**Please Tick (🗸) the applicable box using the below key:**

**1**- Strongly disagree **2**- Disagree **3**- Neutral

**4**- Agree **5**- Strongly agree **6**- Unable to assess

| **S. No** | **Teaching & Learning environment** | **1**  **Strongly**  **disagree** | **2**  **Disagree** | **3**  **Neutral** | **4**  **Agree** | **5**  **Strongly agree** | **6**  **Unable to assess** |
| --- | --- | --- | --- | --- | --- | --- | --- |
| **Q1.** | Encourages students to participate actively in discussions |  |  |  |  |  |  |
| **Q2.** | Stimulates students to bring up problems |  |  |  |  |  |  |
| **Q3.** | Keeps to teaching goals; avoids digressions |  |  |  |  |  |  |
| **Q4.** | Prepares well for teaching presentations and talks |  |  |  |  |  |  |
| **Q5.** | Teaches the topics in theory and practical* |  |  |  |  |  |  |
| **Q6.** | Covering all the points in the topic* |  |  |  |  |  |  |
| **Professional attitude towards students** | | | | | | | |
| **Q7.** | Listens attentively to students |  |  |  |  |  |  |
| **Q8.** | Is respectful towards students |  |  |  |  |  |  |
| **Q9.** | Is available regularly for the students |  |  |  |  |  |  |
| **Q10.** | Is easily approachable for discussions |  |  |  |  |  |  |
| **Communication of Goals** | | | | | | | |
| **Q11.** | States learning goals clearly |  |  |  |  |  |  |
| **Q12.** | Prioritizes learning goals and topics |  |  |  |  |  |  |
| **Q13.** | Debriefing the learning goals periodically |  |  |  |  |  |  |
| **Evaluation of Students** | | | | | | | |
| **Q14.** | Evaluates student’s specialty knowledge regularly |  |  |  |  |  |  |
| **Q15.** | Evaluates student’s analytical abilities regularly |  |  |  |  |  |  |
| **Q16.** | Evaluates student’s application of knowledge to specific patients |  |  |  |  |  |  |
| **Q17.** | Evaluates student’s medical skills regularly |  |  |  |  |  |  |
| **Q18.** | Evaluates student’s, communication and professionalism during patient encounter |  |  |  |  |  |  |
| **Feedback** | | | | | | | |
| **Q19.** | Regularly gives constructive feedbacks to students |  |  |  |  |  |  |
| **Q20.** | Explains why students are incorrect |  |  |  |  |  |  |
| **Q21.** | Offers suggestions for improvement |  |  |  |  |  |  |
| **Q22.** | Gives students chance to reflect on the feedback |  |  |  |  |  |  |
| **Promoting self-directed learning** | | | | | | | |
| **Q23.** | Motivates students to study further and deeper in the topic |  |  |  |  |  |  |
| **Q24.** | Stimulates students to keep up with the literature |  |  |  |  |  |  |
| **Q25.** | Motivates students to learn independently |  |  |  |  |  |  |

*** *Questions 5 and 6 were modified and used in the SETQ: Evaluation of Clinical Tutor by Students.***
