# Supplementary material for: Process assessment of the attitude, ethics, and communication (AETCOM) sessions: student engagement and satisfaction among medical students in central India
Source: BMC Med Educ. 2026 Jun 13;26:963. doi: 10.1186/s12909-026-09691-w (PMC13263919; doi:10.1186/s12909-026-09691-w)
Supplement: Supplementary file 2 — Supplementary Material 2. [file 12909_2026_9691_MOESM2_ESM.docx]

**
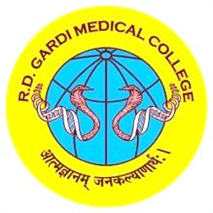

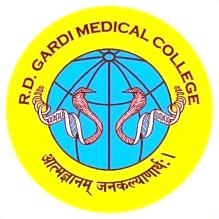
**

**Student engagement checklist**

**Name of observer: ___________________ Topic: ____________________ Date: _______**

| **S.no** | **Time** | **Student Name &**  **Roll No.** | **Eye contact** | **Following instructions** | **On-task behaviour** | **Note taking** | **Distraction (eg. talking, fidgeting, off-task)** |
| --- | --- | --- | --- | --- | --- | --- | --- |
|  |  |  | **Yes/No** | **Yes/No** | **Yes/No** | **Yes/No** | **Yes**  **/No** |
| 1 |  |  |  |  |  |  |  |
| 2 |  |  |  |  |  |  |  |
| 3 |  |  |  |  |  |  |  |
| 4 |  |  |  |  |  |  |  |
| 5 |  |  |  |  |  |  |  |
| 6 |  |  |  |  |  |  |  |
| 7 |  |  |  |  |  |  |  |
| 8 |  |  |  |  |  |  |  |
| 9 |  |  |  |  |  |  |  |
| 10 |  |  |  |  |  |  |  |

**Definitions:**

- **Eye Contact:** **Looking at the teacher** or instructional material.
- **Following Instructions:** **Responds to** directions promptly.
- **On-task behavior:** Engaged with class activity (writing, reading and **participating**).
- **Note-taking:** Writing relevant points or interacting with materials.
- **Distraction:** **Includes talking to peers**, looking away for long periods, playing with objects (mobile scrolling, looking outside in the corridor) etc.

**
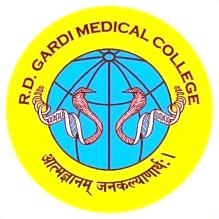

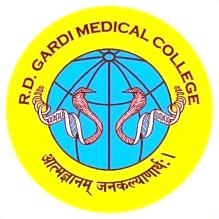
**
