# Supplementary material for: Process assessment of the attitude, ethics, and communication (AETCOM) sessions: student engagement and satisfaction among medical students in central India
Source: BMC Med Educ. 2026 Jun 13;26:963. doi: 10.1186/s12909-026-09691-w (PMC13263919; doi:10.1186/s12909-026-09691-w)
Supplement: Supplementary file 3 — Supplementary Material 3. [file 12909_2026_9691_MOESM3_ESM.docx]

**Supplementary Table 1.** Students’ evaluation of the AETCOM session-2 on the competency: **Disclosure of medical errors**

| S.no | Activities | n | Valuation | | | | | Total score obtains | Possible  maximum score |
| --- | --- | --- | --- | --- | --- | --- | --- | --- | --- |
|  |  |  | 1 | 2 | 3 | 4 | 5 |  |  |
| **Teaching & Learning environment** | |  |  |  |  |  |  |  |  |
| Q1. | Encourages students to participate actively in discussions | 125 | 0 | 0 | 3 | 39 | 83 | 580 | 625 |
| Q2. | Stimulates students to bring up problems | 125 | 1 | 0 | 7 | 45 | 72 | 562 | 625 |
| Q3. | Keeps to teaching goals; avoids digressions | 125 | 4 | 2 | 5 | 50 | 64 | 543 | 625 |
| Q4. | Prepares well for teaching presentations and talks | 125 | 3 | 0 | 5 | 36 | 81 | 567 | 625 |
| Q5. | Teaches the topic in theory and practical | 125 | 1 | 0 | 8 | 38 | 78 | 567 | 625 |
| Q6. | Covering all the points in the topic | 125 | 2 | 0 | 4 | 38 | 81 | 571 | 625 |
| **Professional attitude towards students** | |  |  |  |  |  |  |  |  |
| Q7. | Listens attentively to students | 125 | 2 | 0 | 6 | 45 | 72 | 560 | 625 |
| Q8. | Is respectful towards students | 125 | 2 | 0 | 6 | 39 | 78 | 566 | 625 |
| Q9. | Is available regularly for the students | 125 | 2 | 0 | 9 | 47 | 67 | 552 | 625 |
| Q10. | Is easily approachable for discussions | 125 | 2 | 0 | 7 | 43 | 73 | 560 | 625 |
| **Communication of Goals** | |  |  |  |  |  |  |  |  |
| Q11. | States learning goals clearly | 125 | 2 | 0 | 6 | 45 | 72 | 560 | 625 |
| Q12. | Prioritizes learning goals and topics | 125 | 2 | 0 | 5 | 39 | 79 | 568 | 625 |
| Q13. | Debriefing the learning goals periodically | 125 | 2 | 0 | 9 | 47 | 67 | 552 | 625 |
| **Evaluation of Students** | |  |  |  |  |  |  |  |  |
| Q14. | Evaluates student’s specialty knowledge regularly | 125 | 2 | 0 | 11 | 55 | 57 | 540 | 625 |
| Q15. | Evaluates student’s analytical abilities regularly | 125 | 1 | 0 | 11 | 57 | 55 | 537 | 625 |
| Q16. | Evaluates student’s application of knowledge to specific patients | 125 | 1 | 0 | 14 | 48 | 62 | 545 | 625 |
| Q17. | Evaluates student’s medical skills regularly | 125 | 1 | 0 | 12 | 58 | 53 | 534 | 625 |
| Q18. | Evaluates student’s, communication and professionalism during patient encounter | 125 | 1 | 0 | 7 | 50 | 67 | 557 | 625 |
| **Feedback** | |  |  |  |  |  |  |  |  |
| Q19. | Regularly gives constructive feedbacks to students | 125 | 1 | 1 | 10 | 55 | 58 | 543 | 625 |
| Q20. | Explains why students are incorrect | 125 | 0 | 0 | 7 | 55 | 63 | 556 | 625 |
| Q21. | Offers suggestions for improvement | 125 | 1 | 0 | 9 | 43 | 72 | 560 | 625 |
| Q22. | Gives students chance to reflect on the feedback | 125 | 1 | 0 | 10 | 46 | 68 | 555 | 625 |
| **Promoting self-directed learning** | |  |  |  |  |  |  |  |  |
| Q23. | Motivates students to study further and deeper in the topic | 125 | 1 | 1 | 5 | 51 | 67 | 557 | 625 |
| Q24. | Stimulates students to keep up with the literature | 125 | 1 | 1 | 7 | 48 | 68 | 556 | 625 |
| Q25. | Motivates students to learn independently | 125 | 1 | 1 | 7 | 48 | 68 | 556 | 625 |
|  |  |  | 35 | 6 | 184 | 1188 | 1712 | 13,904 | 15,625 |

Score Calculation**:**

× 100

Total Score obtained

Maximum Possible score

× 100

Total Score obtained

Maximum Possible score

Score (%) =

Total Score obtained

Maximum Possible score

- Total score obtained = 13,904
- Maximum possible score = 25 questions × 125 respondents × 5 = 15,625

13,904

15,625

Score (%) =

× 100 = 88.99%

(very good)
