# Supplementary material for: Process assessment of the attitude, ethics, and communication (AETCOM) sessions: student engagement and satisfaction among medical students in central India
Source: BMC Med Educ. 2026 Jun 13;26:963. doi: 10.1186/s12909-026-09691-w (PMC13263919; doi:10.1186/s12909-026-09691-w)
Supplement: Supplementary file 4 — Supplementary Material 4. [file 12909_2026_9691_MOESM4_ESM.docx]

**Supplementary Table 2.** Students’ evaluation of the AETCOM session-3 on the competency: **Confidentiality in patient’s care**

| S.no | Activities | n | Valuation | | | | | Total score obtain | Possible  maximum score |
| --- | --- | --- | --- | --- | --- | --- | --- | --- | --- |
|  |  |  | 1 | 2 | 3 | 4 | 5 |  |  |
| **Teaching & Learning environment** | |  |  |  |  |  |  |  |  |
| Q1. | Encourages students to participate actively in discussions | 118 | 0 | 0 | 2 | 54 | 62 | 532 | 590 |
| Q2. | Stimulates students to bring up problems | 118 | 1 | 0 | 5 | 46 | 66 | 530 | 590 |
| Q3. | Keeps to teaching goals; avoids digressions | 118 | 0 | 0 | 4 | 50 | 64 | 532 | 590 |
| Q4. | Prepares well for teaching presentations and talks | 118 | 0 | 0 | 3 | 43 | 72 | 541 | 590 |
| Q5. | Teaches the topic in theory and practical | 118 | 1 | 0 | 2 | 51 | 64 | 531 | 590 |
| Q6. | Covering all the points in the topic | 118 | 1 | 0 | 7 | 37 | 73 | 535 | 590 |
| **Professional attitude towards students** | |  |  |  |  |  |  |  |  |
| Q7. | Listens attentively to students | 118 | 1 | 0 | 4 | 44 | 69 | 534 | 590 |
| Q8. | Is respectful towards students | 118 | 0 | 0 | 2 | 48 | 68 | 538 | 590 |
| Q9. | Is available regularly for the students | 118 | 1 | 0 | 5 | 47 | 65 | 529 | 590 |
| Q10. | Is easily approachable for discussions | 118 | 0 | 0 | 6 | 43 | 69 | 535 | 590 |
| **Communication of Goals** | |  |  |  |  |  |  |  |  |
| Q11. | States learning goals clearly | 118 | 0 | 0 | 5 | 48 | 65 | 532 | 590 |
| Q12. | Prioritizes learning goals and topics | 118 | 0 | 0 | 2 | 53 | 63 | 533 | 590 |
| Q13. | Debriefing the learning goals periodically | 118 | 0 | 0 | 4 | 52 | 62 | 530 | 590 |
| **Evaluation of Students** | |  |  |  |  |  |  |  |  |
| Q14. | Evaluates student’s specialty knowledge regularly | 118 | 0 | 0 | 10 | 52 | 56 | 518 | 590 |
| Q15. | Evaluates student’s analytical abilities regularly | 118 | 0 | 0 | 8 | 53 | 57 | 521 | 590 |
| Q16. | Evaluates student’s application of knowledge to specific patients | 118 | 0 | 0 | 9 | 48 | 61 | 524 | 590 |
| Q17. | Evaluates student’s medical skills regularly | 118 | 0 | 0 | 6 | 53 | 59 | 525 | 590 |
| Q18. | Evaluates student’s, communication and professionalism during patient encounter | 118 | 0 | 0 | 6 | 51 | 61 | 527 | 590 |
| **Feedback** | |  |  |  |  |  |  |  |  |
| Q19. | Regularly gives constructive feedbacks to students | 118 | 0 | 0 | 8 | 52 | 58 | 522 | 590 |
| Q20. | Explains why students are incorrect | 118 | 0 | 0 | 7 | 51 | 60 | 525 | 590 |
| Q21. | Offers suggestions for improvement | 118 | 0 | 0 | 4 | 51 | 63 | 531 | 590 |
| Q22. | Gives students chance to reflect on the feedback | 118 | 0 | 0 | 3 | 52 | 63 | 532 | 590 |
| **Promoting self-directed learning** | |  |  |  |  |  |  |  |  |
| Q23. | Motivates students to study further and deeper in the topic | 118 | 0 | 1 | 7 | 44 | 66 | 529 | 590 |
| Q24. | Stimulates students to keep up with the literature | 118 | 0 | 0 | 6 | 44 | 68 | 534 | 590 |
| Q25. | Motivates students to learn independently | 118 | 0 | 0 | 5 | 46 | 67 | 534 | 590 |
|  |  |  | 5 | 1 | 130 | 1213 | 1601 | 13,254 | 14,750 |

Score Calculation:

× 100

Total Score obtained

Maximum Possible score

Score (%) =

- Total score obtained = 13,254
- Maximum possible score = 25 questions × 118 respondents × 5 = 14,750

13,254

14,750

× 100 = 89.86%

(very good)

Score (%) =
