# Supplementary material for: Process assessment of the attitude, ethics, and communication (AETCOM) sessions: student engagement and satisfaction among medical students in central India
Source: BMC Med Educ. 2026 Jun 13;26:963. doi: 10.1186/s12909-026-09691-w (PMC13263919; doi:10.1186/s12909-026-09691-w)
Supplement: Supplementary file 5 — Supplementary Material 5. [file 12909_2026_9691_MOESM5_ESM.docx]

**Supplementary Table 3.** Students’ evaluation of the AETCOM session-4 on the competency: **Fiduciary duty of a doctor**

| S.no | Activities | n | Valuation | | | | | Total score obtain | Possible  maximum score |
| --- | --- | --- | --- | --- | --- | --- | --- | --- | --- |
|  |  |  | 1 | 2 | 3 | 4 | 5 |  |  |
| **Teaching & Learning environment** | |  |  |  |  |  |  |  |  |
| Q1. | Encourages students to participate actively in discussions | 113 | 0 | 0 | 4 | 47 | 62 | 510 | 565 |
| Q2. | Stimulates students to bring up problems | 113 | 0 | 0 | 7 | 48 | 58 | 503 | 565 |
| Q3. | Keeps to teaching goals; avoids digressions | 113 | 0 | 0 | 1 | 51 | 61 | 512 | 565 |
| Q4. | Prepares well for teaching presentations and talks | 113 | 0 | 0 | 2 | 45 | 66 | 516 | 565 |
| Q5. | Teaches the topic in theory and practical | 113 | 0 | 0 | 2 | 49 | 62 | 512 | 565 |
| Q6. | Covering all the points in the topic | 113 | 0 | 0 | 3 | 38 | 72 | 521 | 565 |
| **Professional attitude towards students** | |  |  |  |  |  |  |  |  |
| Q7. | Listens attentively to students | 113 | 0 | 0 | 5 | 41 | 67 | 514 | 565 |
| Q8. | Is respectful towards students | 113 | 0 | 0 | 4 | 51 | 58 | 506 | 565 |
| Q9. | Is available regularly for the students | 113 | 0 | 0 | 4 | 44 | 65 | 513 | 565 |
| Q10. | Is easily approachable for discussions | 113 | 0 | 0 | 4 | 45 | 64 | 512 | 565 |
| **Communication of Goals** | |  |  |  |  |  |  |  |  |
| Q11. | States learning goals clearly | 113 | 0 | 0 | 6 | 53 | 54 | 500 | 565 |
| Q12. | Prioritizes learning goals and topics | 113 | 0 | 0 | 5 | 47 | 61 | 508 | 565 |
| Q13. | Debriefing the learning goals periodically | 113 | 0 | 0 | 3 | 49 | 61 | 510 | 565 |
| **Evaluation of Students** | |  |  |  |  |  |  |  |  |
| Q14. | Evaluates student’s specialty knowledge regularly | 113 | 0 | 1 | 11 | 54 | 47 | 486 | 565 |
| Q15. | Evaluates student’s analytical abilities regularly | 113 | 0 | 0 | 9 | 48 | 56 | 499 | 565 |
| Q16. | Evaluates student’s application of knowledge to specific patients | 113 | 0 | 1 | 7 | 54 | 51 | 494 | 565 |
| Q17. | Evaluates student’s medical skills regularly | 113 | 0 | 1 | 6 | 48 | 58 | 502 | 565 |
| Q18. | Evaluates student’s, communication and professionalism during patient encounter | 113 | 0 | 0 | 6 | 43 | 64 | 510 | 565 |
| **Feedback** | |  |  |  |  |  |  |  |  |
| Q19. | Regularly gives constructive feedbacks to students | 113 | 0 | 0 | 6 | 55 | 52 | 498 | 565 |
| Q20. | Explains why students are incorrect | 113 | 0 | 0 | 7 | 48 | 58 | 503 | 565 |
| Q21. | Offers suggestions for improvement | 113 | 0 | 0 | 4 | 51 | 58 | 506 | 565 |
| Q22. | Gives students chance to reflect on the feedback | 113 | 0 | 0 | 8 | 41 | 64 | 508 | 565 |
| **Promoting self-directed learning** | |  |  |  |  |  |  |  |  |
| Q23. | Motivates students to study further and deeper in the topic | 113 | 0 | 0 | 5 | 52 | 56 | 503 | 565 |
| Q24. | Stimulates students to keep up with the literature | 113 | 0 | 0 | 5 | 47 | 61 | 508 | 565 |
| Q25. | Motivates students to learn independently | 113 | 0 | 0 | 2 | 38 | 73 | 523 | 565 |
|  |  |  | 0 | 3 | 116 | 1207 | 1499 | 12,677 | 14,125 |

Score Calculation**:**

× 100

Total Score obtained

Maximum Possible score

Score (%) =

- Total score obtained = 12,677
- Maximum possible score = 25 questions × 113 respondent × 5 = 14,125

12,677

14,125

× 100 = 89.73%

(very good)

Score (%) =
